# [Retracted] Fisetin inhibits migration, invasion and epithelial-mesenchymal transition of LMP1-positive nasopharyngeal carcinoma cells

**DOI:** 10.3892/mmr.2025.13628

**Published:** 2025-07-22

**Authors:** Rong Li, Yinhai Zhao, Jin Chen, Songjun Shao, Xiujuan Zhang

Mol Med Rep 9: 413–418, 2014; DOI: 10.3892/mmr.2013.1836

Following the publication of the above paper, it was drawn to the Editor's attention by a concerned reader that the cell migration and invasion assay data shown in Fig. 1A and B on p. 415 contained a number of overlapping data sections, such that data which were intended to show the results from differently performed experiments had apparently been derived from the same original sources.

In view of the large number of panels containing overlapping sections of data that were identified in Fig. 1, the Editor of *Molecular Medicine Reports* has decided that this paper should be retracted from the Journal due to a lack of confidence in the presented data. The authors were asked for an explanation to account for these concerns, but the Editorial Office did not receive a reply. The Editor apologizes to the readership for any inconvenience caused.

